# Supplementary figures and images for: Strong Selection Significantly Increases Epistatic Interactions in the Long-Term Evolution of a Protein
Source: PLoS Genet. 2016 Mar 30;12(3):e1005960. doi: 10.1371/journal.pgen.1005960 (PMC4814079; doi:10.1371/journal.pgen.1005960)

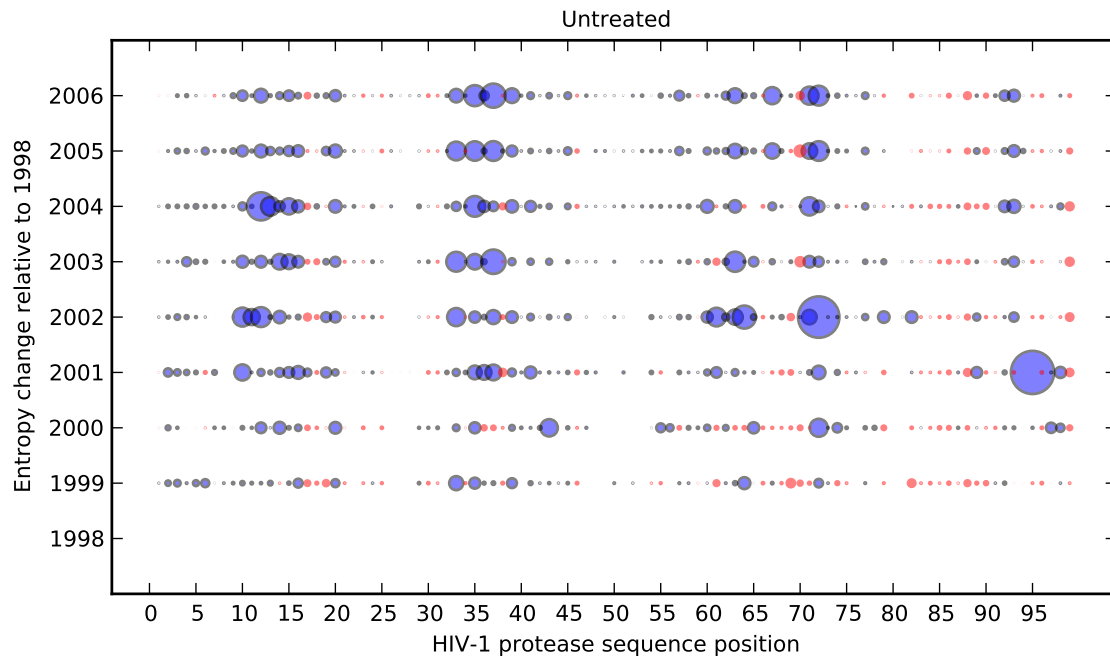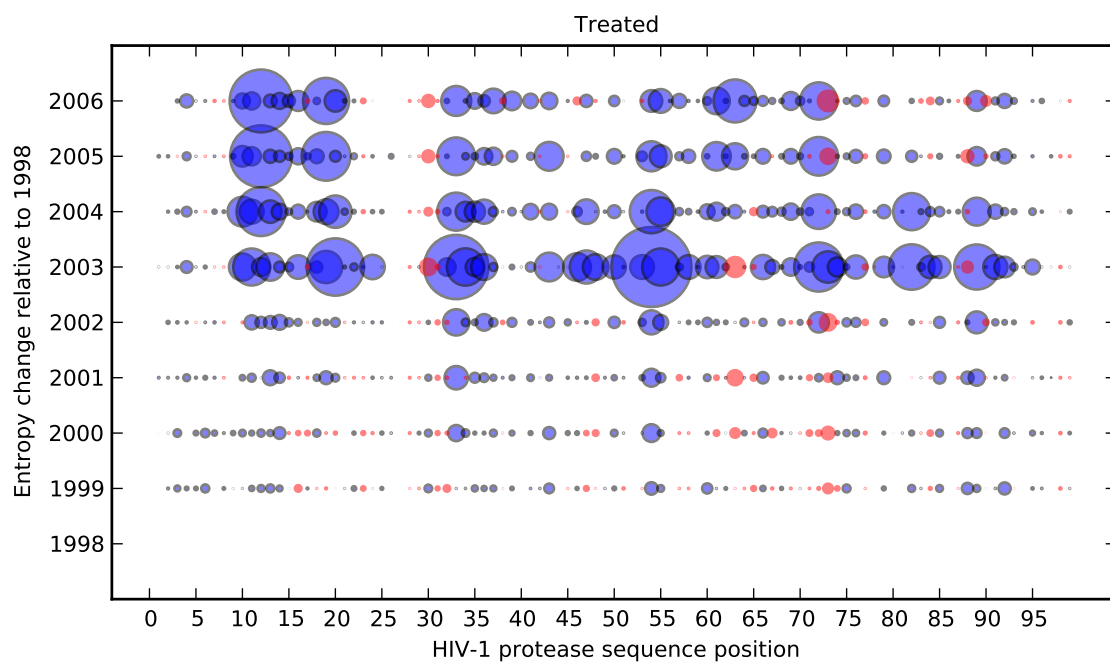

Supplement: S1 Fig — Average entropy change (compared to 1998) at every position of the HIV-1 protease in the untreated (top panel) and treated (bottom panel) data sets. The size of the circles is proportional to the entropy change, and blue marks an increase while red implies a decrease in entropy at that site, compared to 1998 (the first year in our analysis). Site-specific variation mostly increased across the protein even in the absence of treatment, but decreased at some sites. In the treated data set, the entropy increased at most sites (in particular starting in 2003) while some sites became less entropic. (PDF) [file pgen.1005960.s001.pdf]

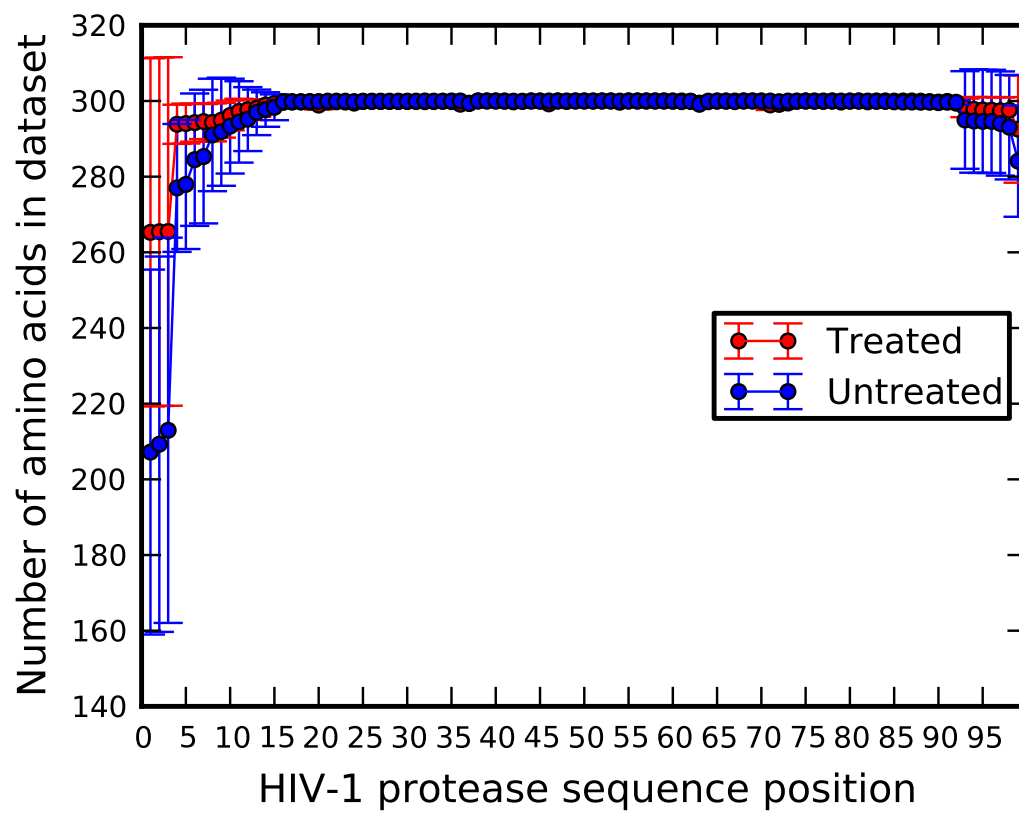

Supplement: S2 Fig — Gaps at the beginning and ends of the protease sequence imply uneven sample size for positions ≥15 and ≤ 90, and thus the ends were truncated for calculation of per-site entropies and pairwise mutual information. Filled circles represent average number of residues in the sampled sets at each protease position and error bars represent unit SD. (PDF) [file pgen.1005960.s002.pdf]
