# Supplementary material for: Strong Selection Significantly Increases Epistatic Interactions in the Long-Term Evolution of a Protein
Source: PLoS Genet. 2016 Mar 30;12(3):e1005960. doi: 10.1371/journal.pgen.1005960 (PMC4814079; doi:10.1371/journal.pgen.1005960)
Supplement: S1 Table — (PDF) [file pgen.1005960.s003.pdf]

Supporting Table S1. Protease Inhibitors (PIs) and the years they were approved by FDA. New drugs with higher genetic barriers to resistance are continually developed to provide treatment options to patients showing resistance to earlier drugs.

Drug (PI)	Year	Comment
Saquinavir	1996	1st-line PI, given with Ritonavir
Indinavir	1996	1st-line PI. Metabolized quickly, requiring multiple doses per day to avoid drug resistance
Ritonavir	1996	Initially used as a 1st-line PI, now used in combination with other PIs due to its ability to metabolize the enzyme that metabolizes other PIs
Nelfinavir	1997	1st-line PI
Amprenavir	1999	Discontinued in 2004, prodrug version still available (Fosamprenavir)
Lopinavir	2000	2nd-line PI, given with Ritonavir in patients with some drug resistance
Atazanavir	2003	2nd-line PI, given with Ritonavir in patients with some drug resistance
Fosamprenavir	2003	Slow-release version of Amprenavir
Tipranavir	2005	2nd-line PI, given with Ritonavir in patients with some drug resistance
Darunavir	2006	2nd-line PI, given with Ritonavir in patients with some drug resistance
