# Supplementary material for: Strong Selection Significantly Increases Epistatic Interactions in the Long-Term Evolution of a Protein
Source: PLoS Genet. 2016 Mar 30;12(3):e1005960. doi: 10.1371/journal.pgen.1005960 (PMC4814079; doi:10.1371/journal.pgen.1005960)
Supplement: S1 Text — (PDF) [file pgen.1005960.s004.pdf]

Supporting Text S1: Phylogenetic structure of protease sequence data

We obtained protease sequences from the HIV Stanford Database [1, 2] that compiles data from several different sources. Although it has been shown that due to the high mutation rate of HIV-1, most amino acid covariation results from selection pressure and not linkage disequilibrium [3], we constructed the phylogenetic trees to rule out any confounding phylogenetic effects [4]. Only trees for even years are shown, although data from all years show similar star-like phylogeny.

Since we resampled 300 sequences from sequences available for each year and environment (treated and untreated), we show neighbor joining trees for each of these resampled datasets. The figures were made using the phylogeny inference package Phylip, where we used program *protdist* to compute distances between all pairs of sequences for constructing the neighbor joining trees [5]. All the trees show star-phylogeny, ruling out subdivisions in the data due to phylogenetic effects. The trees made with 100 bootstraps also showed star-like phylogenies (data not shown).

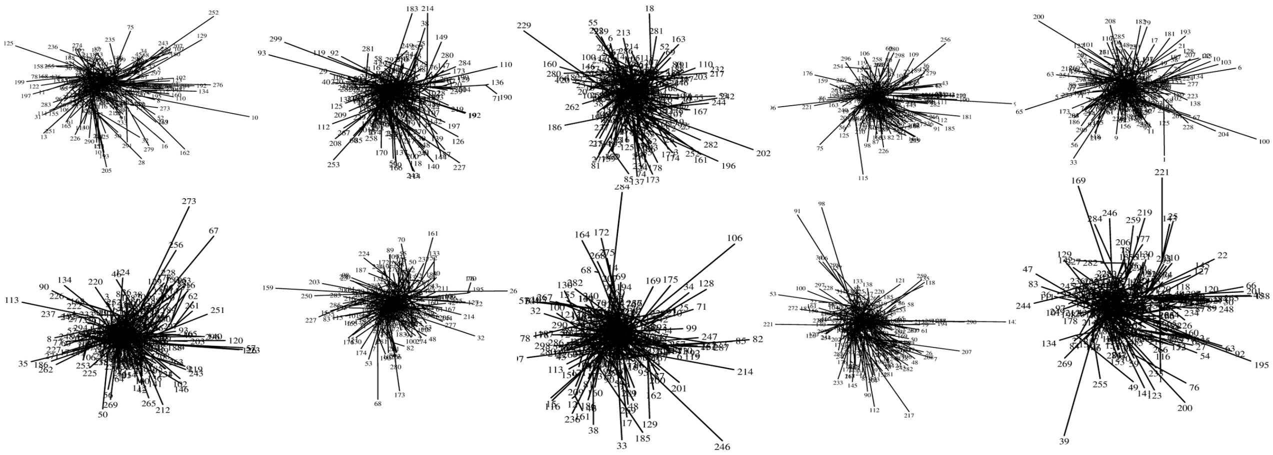

Phylogenies of subsampled datasets from year 1998 (untreated sequences)

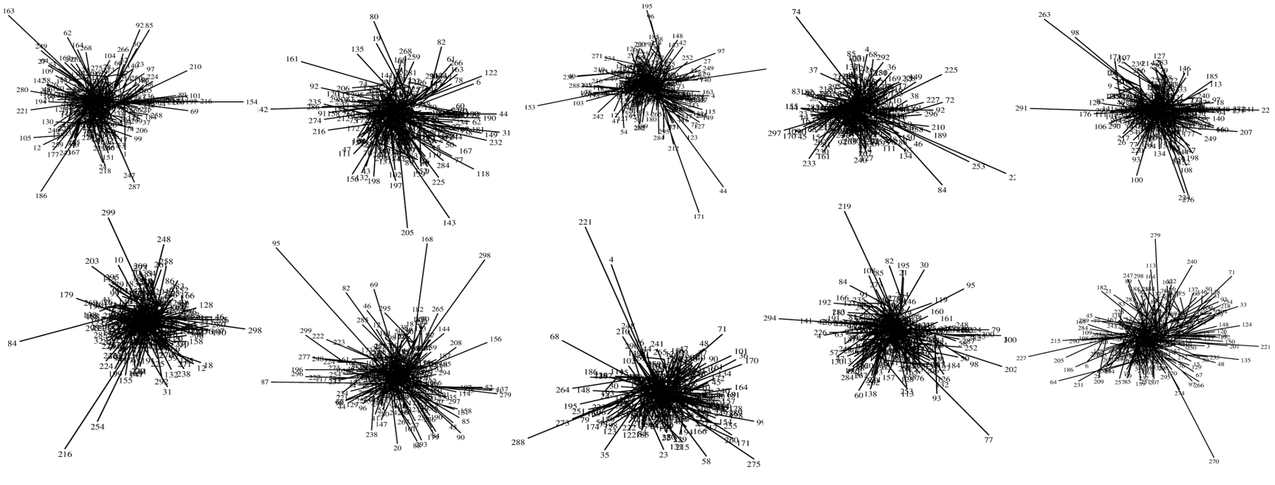

Phylogenies of subsampled datasets from year 2000 (untreated sequences)

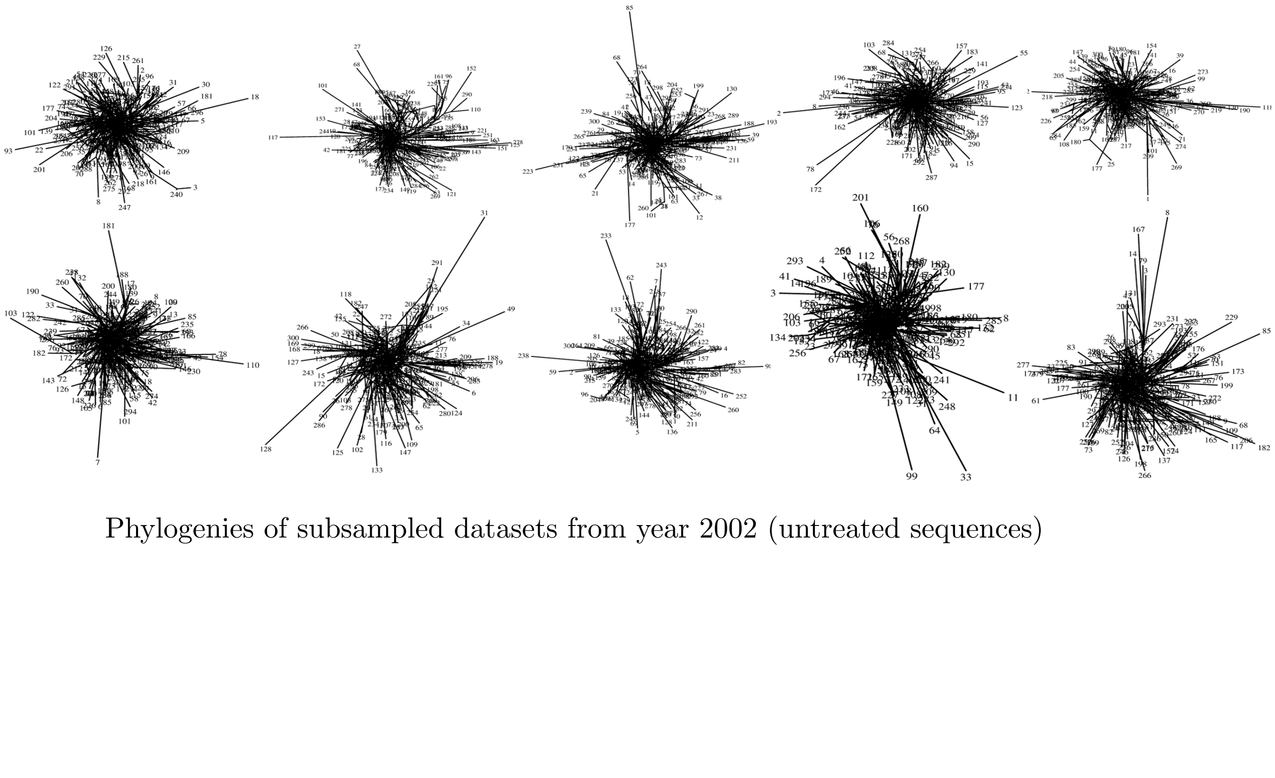

Phylogenies of subsampled datasets from year 2002 (untreated sequences)

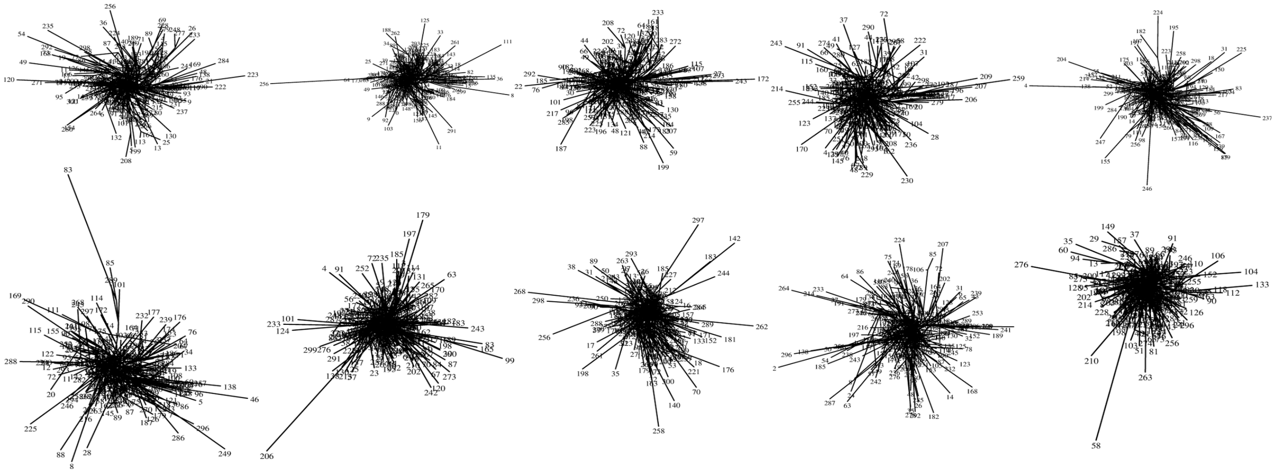

Phylogenies of subsampled datasets from year 2004 (untreated sequences)

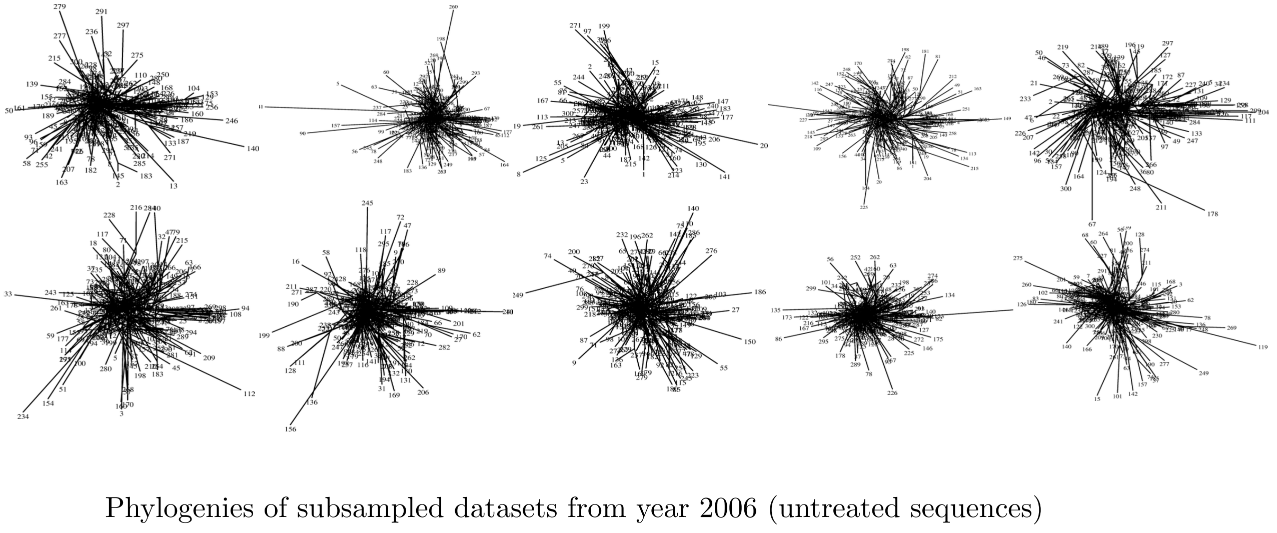

Phylogenies of subsampled datasets from year 2006 (untreated sequences)

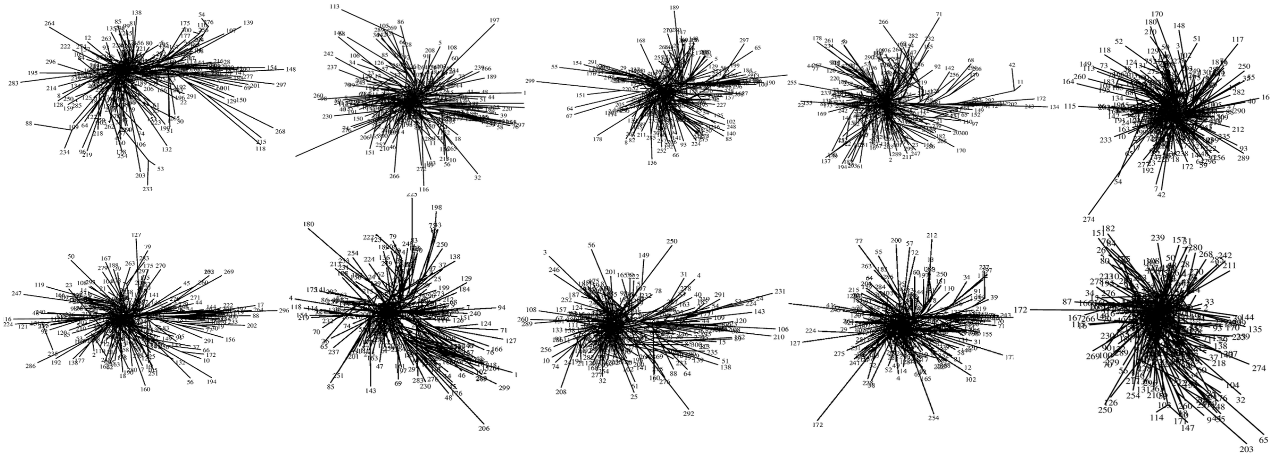

Phylogenies of subsampled datasets from year 1998 (treated sequences)

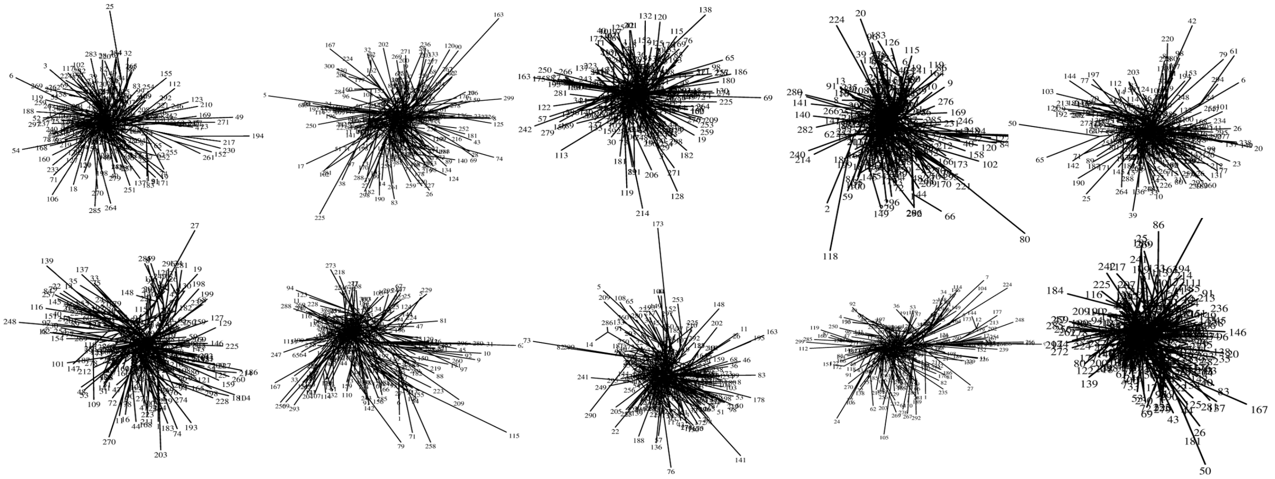

Phylogenies of subsampled datasets from year 2000 (treated sequences)

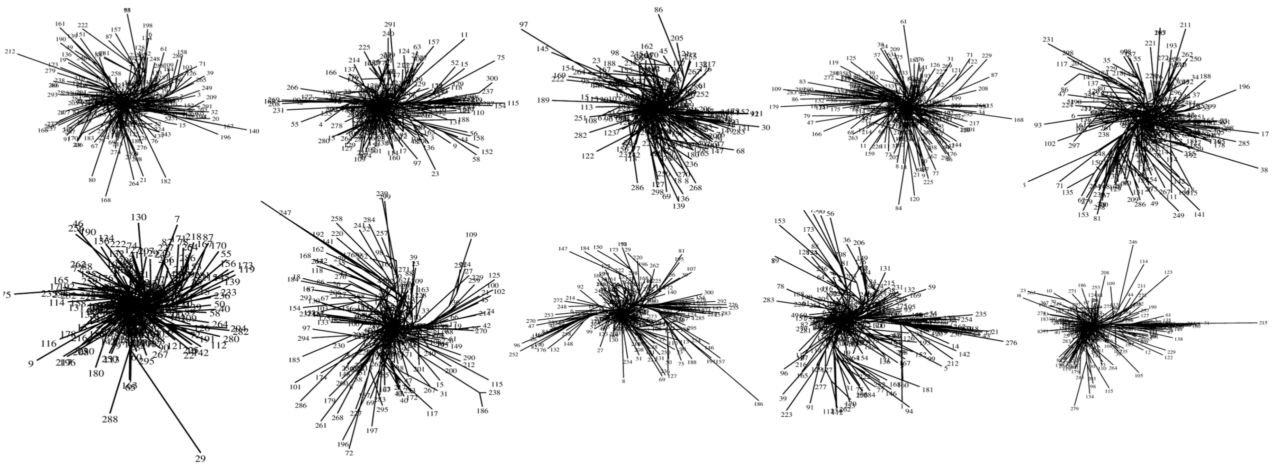

Phylogenies of subsampled datasets from year 2002 (treated sequences)

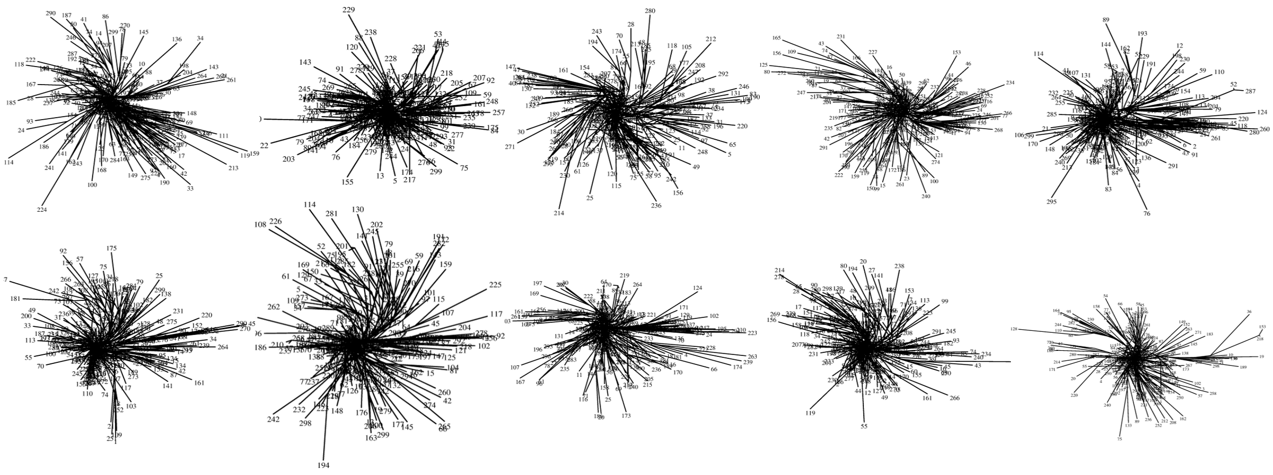

Phylogenies of subsampled datasets from year 2004 (treated sequences)

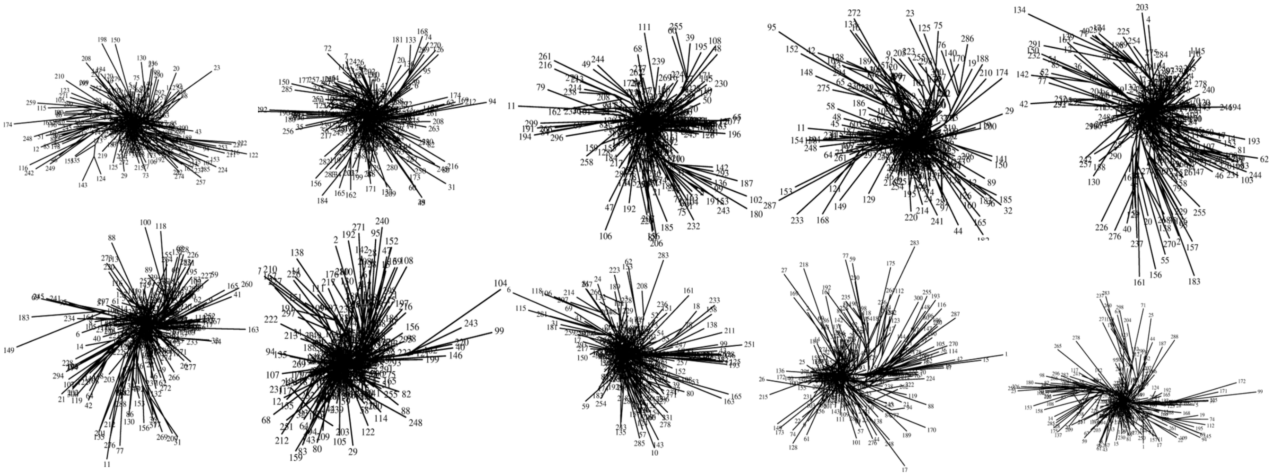

Phylogenies of subsampled datasets from year 2006 (treated sequences)

References

[1] Rhee SY, Gonzales MJ, Kantor R, Betts BJ, Ravela J, Shafer RW. Human immunodeficiency virus reverse transcriptase and protease sequence database. Nucleic Acids Res. 2003;31:298–303.

[2] Shafer RW. Rationale and uses of a public HIV drug-resistance database. J Infect Dis. 2006;194 Suppl 1:S51–8.

[3] Wang Q, Lee C. Distinguishing functional amino acid covariation from background linkage disequilibrium in HIV protease and reverse transcriptase. PLoS One. 2007;2:e814.

[4] Lunzer M, Golding GB, Dean AM. Pervasive cryptic epistasis in molecular evolution. PLoS Genet. 2010;6:e1001162.

[5] Felsenstein J. PHYLIP- Phylogeny Inference Package. Cladistics. 1989;5:164–166.
