# Supplementary material for: Strong Selection Significantly Increases Epistatic Interactions in the Long-Term Evolution of a Protein
Source: PLoS Genet. 2016 Mar 30;12(3):e1005960. doi: 10.1371/journal.pgen.1005960 (PMC4814079; doi:10.1371/journal.pgen.1005960)
Supplement: S2 Text — (PDF) [file pgen.1005960.s005.pdf]

Supporting Text S2: Sequence Logos for HIV-1 Protease treated and untreated sequences.

Sequence logos from our treated and untreated HIV-1 protease data from all years are shown below (logos generated using Weblogo [1]). The residues are colored according to their chemical properties: polar residues as green; neutral residues as purple; basic residues as blue; acidic residues as red; and hydrophobic residues as black.

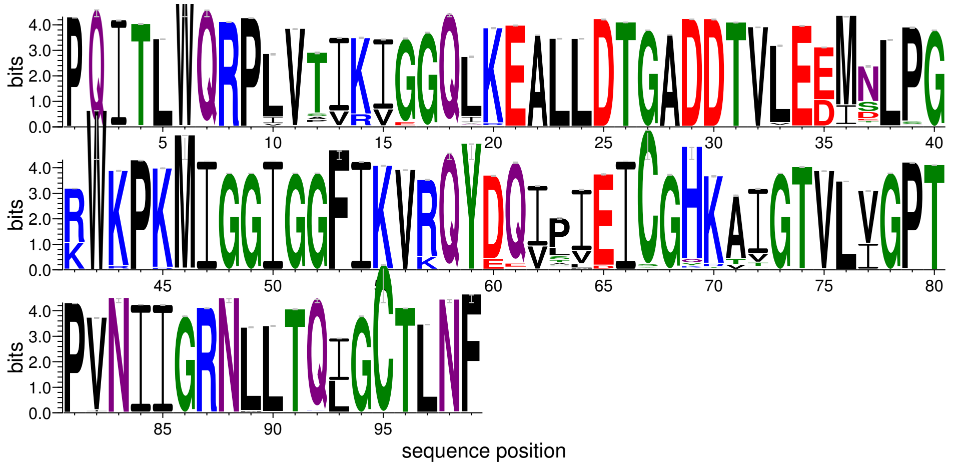

Sequence logo for the untreated protease sequences from all years.

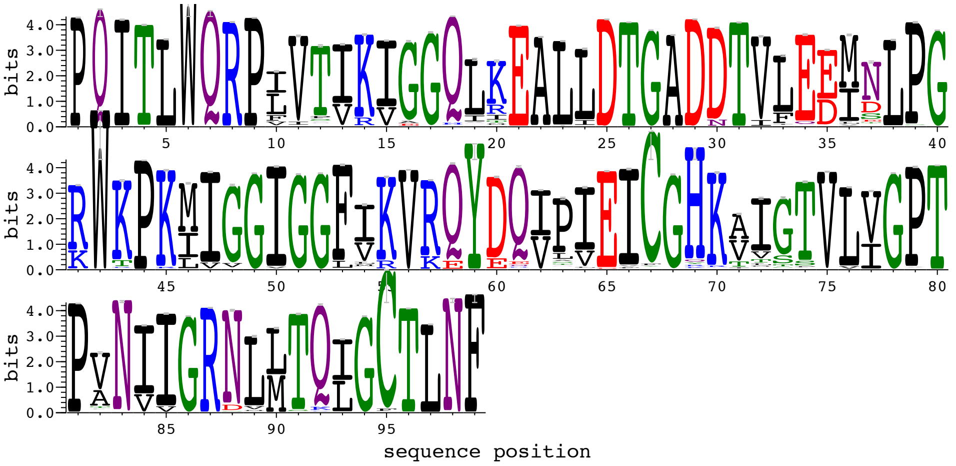

Sequence logo for the treated protease sequences from all years.

References

[1] Crooks GE, Hon G, Chandonia JM, Brenner SE. WebLogo: a sequence logo generator. Genome Res. 2004;14:1188–1190.
