# Supplementary material for: Strong Selection Significantly Increases Epistatic Interactions in the Long-Term Evolution of a Protein
Source: PLoS Genet. 2016 Mar 30;12(3):e1005960. doi: 10.1371/journal.pgen.1005960 (PMC4814079; doi:10.1371/journal.pgen.1005960)
Supplement: S3 Text — (PDF) [file pgen.1005960.s006.pdf]

Supporting Text S3: Changes in physico-chemical properties at protease positions mirror per-site entropy changes

Our analysis of residue properties show that the increase in entropy is often associated with changes in residue charge or size at that position (Figure 2 main text). The "isoelectric point" (pI) is the pH at which there is no charge on the residue: positively charged residues have a higher pI (Arg R: 10.76, Lys K: 9.74) and negatively charged residues have a low pI (Asp acid D: 2.77, Glu acid E: 3.22, see Table below for pI and residue-weight of all residues). A negative pI difference implies that residues with a higher isoelectric point (more positively charged, thus more basic) are replaced by acidic and negatively charged residues upon treatment (Figure 2, main text, middle panel). Positive pI difference means that position is becoming more basic upon treatment. For position 30, although the entropy increase is not as significant as that at other loci, the loss of negative charge at the position (mutation from aspartic acid to asparagine, a major drug-resistance mutation) is substantial (Figure 2, main text, middle panel). Position 20, on the other hand, shows a decrease in pI following treatment, suggesting that the position has become more acidic.

Several protease positions also show a marked shift in residue-weight at positions post-treatment. The residue-weight difference at a position is positive when heavier residues occupy that position after treatment, as is the case for positions 71 (emergence of valine in place of alanine: A71V), 73 (serine is preferred to glycine in some treated sequences: G73S), and 90 (methionine replaces leucine in 50% of treated sequences, L90M; Figure 2, bottom panel in main text). These heavier residues seen in treated protease sequences are also larger in size than the residues in untreated sequences, and thus could create potential steric clashes unless compensatory mutations occur elsewhere. We also observe negative residue-weight differences at positions 36, 46, 54, and 83, suggesting that smaller residues are now occupying these positions to either avoid steric clashes due to other changes in the protein (accessory mutations), or to change the protein-drug interaction (resistance causing mutations).

Physico-chemical properties of residues

Residue	Isoelectric point (pI)	Residue weight*
A	6.0	71.08
C	5.07	103.15
E	3.22	129.12
D	2.77	115.09
G	5.97	57.05
F	5.48	147.18
I	6.02	113.16
H	7.59	137.14
K	9.74	128.18
M	5.74	131.2
L	5.98	113.16
N	5.41	114.11
Q	5.65	128.13
P	6.3	97.12
S	5.68	87.08
R	10.76	156.19
T	5.6	101.11
W	5.89	186.22
V	5.96	99.13
Y	5.66	163.18

*Residue weight = Molecular weight of amino acid - H_2O

Reference: D.R.Lide, Handbook of Chemistry and Physics, 72nd Edition, CRC Press, Boca Raton, FL, 1991.

http://www.sigmaaldrich.com/life-science/metabolomics/learning-center/amino-acid-reference-chart.html
