# Supplementary material for: Strong Selection Significantly Increases Epistatic Interactions in the Long-Term Evolution of a Protein
Source: PLoS Genet. 2016 Mar 30;12(3):e1005960. doi: 10.1371/journal.pgen.1005960 (PMC4814079; doi:10.1371/journal.pgen.1005960)
Supplement: S4 Text — (PDF) [file pgen.1005960.s007.pdf]

Supporting Text S4: Changes in epistatic interactions in a longitudinal study

The information content trends we highlighted in Figure 3 of the main text are obtained from pooled data taken from many different subjects. To test whether the trends also hold within patients, we analyzed the changes in information content and epistasis from patients enrolled in a longitudinal study. These data (HIV Stanford database, see Methods, protease sequences derived from the same patient at two time-points: first and second isolate) further support the observation that treatment decreases I_1 but increases the sum of pairwise mutual information, *i.e.*, treatment increases per-site variability as well as the extent of epistatic interactions in the protein. For patients that went from 'untreated' to 'treated' state, as well as patients that continued treatment, I_1 showed a slight decrease in the latter isolate.

Stopping treatment shows a slight increase in I_1, suggesting that entropy (amino acid variation) decreases when the selection pressure of drugs is removed, however, this observation is based on a small sample size (153 sequences in 'treated to untreated' category). The sum of pairwise mutual information also increases when treatment is initiated or continued, suggesting that information is redistributed towards interactions upon treatment. We note that while the longitudinal data agrees with the temporal trends for I_1 and I_2, it does not add statistical power to those conclusions due to small sample sizes.

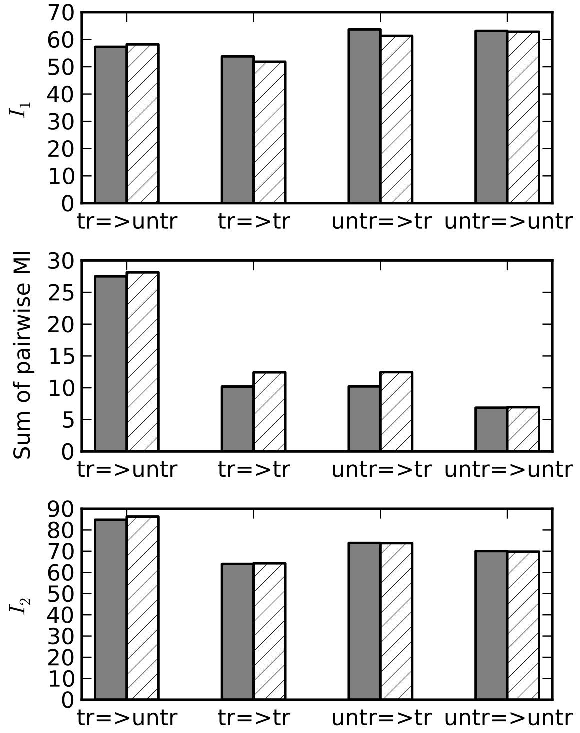

Information measures from longitudinal data. The categories are treated to untreated (tr=>untr), treated to treated (tr=>tr), untreated to treated (untr=>tr), and untreated to untreated (untr=>untr). Grey and hatched bars represent the first and second time-point, respectively. I_1 decreases as entropy increases due to treatment (top panel), while sum of pairwise mutual information increases due to treatment (middle panel). The net information content of the protein, as approximated by I_2, does not show any change in the two isolates collected from the same patient.
