# Supplementary material for: Strong Selection Significantly Increases Epistatic Interactions in the Long-Term Evolution of a Protein
Source: PLoS Genet. 2016 Mar 30;12(3):e1005960. doi: 10.1371/journal.pgen.1005960 (PMC4814079; doi:10.1371/journal.pgen.1005960)
Supplement: S5 Text — (PDF) [file pgen.1005960.s008.pdf]

Supporting Text S5: Information between epistatic pairs in protease of treated and untreated subjects, for spatially close and distant epistatic residue pairs

We analyze the redistribution of information between epistatic pairs over time, separating out spatially close from distant residues. We show the cumulative distribution of information as a function of $\log_{10}$ of information, separately for close residues (those that are thought to be in contact) and those that are distant from each other. As criterion for "closeness" we use a cutoff of $8\AA$ distance between residues in the 3D structure, as is usual in the literature for contact prediction [1, 2, 3]. We separately analyze the interacting pairs within the molecule for early, medium, and late time points as well as for treated and untreated groups (using a larger cutoff such as $10\AA$ does not change the trend).

Inter-residue distances were determined using Bio.PDB, a biopython module for analysis of crystallographic structures [4]. Since the protease is a dimer but the sequence data is that of a single chain, we assume that both chains are identical and compute distances between residues from protease chain A in PDB structure 1F7A [5, 6].

For the sequences from 1998 (left panel), there was little difference in treated and untreated subjects in the distribution of information for close pairs (contacts), but significant difference in the information for distant pairs: the untreated subjects were storing more information in distant pairs than the treated subjects. This trend is reversed already in the year 2002 (middle panel), and more significantly in 2006 (right panel). For those years, molecules under treatment stored more information in pairs than those not under selection, but the significant difference between the trend in close vs. distant residues has all but disappeared by 2006, meaning that the interactions between residues over long-distances (see for example in Fig. 5 of the main text, which have also been observed in other proteins [3]) have disappeared both in the treated as well as the untreated subjects. It is not clear if the effect is solely due to the long-term evolution of the protease or due to complex treatment histories of patients on antiviral therapies.

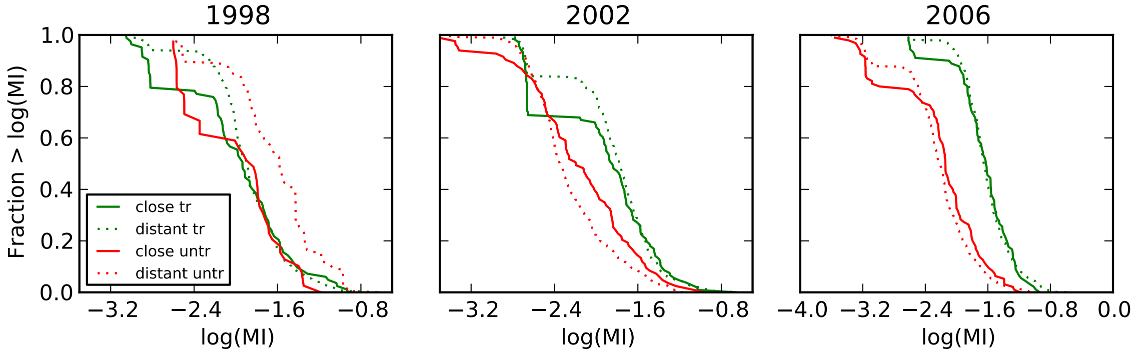

Cumulative distribution of epistatic information for the years 1998, 2002, and 2006. Information in pairs that are in-contact (residue distance $< 8\mathring{A}$), labeled 'close', solid lines), and distant residues (dashed lines, residue distance $\geq 8\mathring{A}$) as a function of the $\log_{10}$ of the mutual information of the pairs. Cumulative distribution for treated subjects in green, for untreated subjects in red. Only pairs with significant mutual information are included.

References

[1] Fodor AA, Aldrich RW. Influence of conservation on calculations of amino acid covariance in multiple sequence alignments. Proteins. 2004; 56:211–21.

[2] Shackelford G, Karplus K. Contact prediction using mutual information and neural nets. Proteins. 2007; 69 Suppl 8:159–64.

[3] Burger L, van Nimwegen E. Disentangling direct from indirect co-evolution of residues in protein alignments. PLoS Comput Biol. 2010; 6:e1000633.

[4] Cock PJA, Antao T, Chang JT, Chapman BA, Cox CJ, Dalke A, et al. Biopython: freely available Python tools for computational molecular biology and bioinformatics. Bioinformatics. 2009; 25:1422–3.

[5] Bernstein FC, Koetzle TF, Williams GJ, Meyer EF Jr, Brice MD, Rodgers JR, et al. The Protein Data Bank: a computer-based archival file for macromolecular structures. Arch Biochem Biophys. 1978; 185:584–91.

[6] Prabu-Jeyabalan M, Nalivaika E, Schiffer CA. How does a symmetric dimer recognize an asymmetric substrate? A substrate complex of HIV-1 protease. J Mol Biol. 2000; 301:1207–20.
