# Supplementary material for: Strong Selection Significantly Increases Epistatic Interactions in the Long-Term Evolution of a Protein
Source: PLoS Genet. 2016 Mar 30;12(3):e1005960. doi: 10.1371/journal.pgen.1005960 (PMC4814079; doi:10.1371/journal.pgen.1005960)
Supplement: S6 Text — (PDF) [file pgen.1005960.s009.pdf]

Supporting Text S6: Correction for sample-size bias in entropy and mutual information estimates

It is well-known that entropy and information estimations from frequencies (maximum-likelihood estimators) are unreliable when sample sizes are small, due to under-sampled probability distributions [1]. Because total information estimation sums over $n(n-1)/2$ residue pairs, it is important to correctly estimate entropies so as not to accumulate finite sample-size errors. We tested several estimators for entropy and, in particular, information calculations:

Maximum likelihood (ML) estimator: empirical values of entropy calculated from observed frequencies.

Miller Madow (MM) estimator: bias-corrected empirical entropy estimator [2].

Jeffreys estimator: Bayesian estimates of the bin frequencies using the Dirichlet-multinomial pseudocount model (pseudocount = 1/2) [3].

Laplace's prior: Bayesian estimates of the bin frequencies using the Dirichlet-multinomial pseudocount model (pseudocount = 1).

SG estimator: Bayesian estimates of the bin frequencies using the Dirichlet-multinomial pseudocount model, pseudocount = 1/20 (since 20 amino acids) [4].

Minimax estimator: Bayesian estimates of the bin frequencies using the Dirichlet-multinomial pseudocount model, pseudocount = $\sqrt{n}/20$ (n = number of sequences, 20 because of 20 possible residues at each position).

Chao Shen (CS) estimator: Proposed by Chao and Shen in 2003 [5].

Shrink estimator: Proposed by Hausser and Strimmer in 2009 [6].

NSB estimator: Proposed by Nemenman, Shafee, and Bialek [7].

To compare the performance of these estimators as sample size is increasing, we computed entropies at positions 54 and 82 of HIV-1 protease (a pair that is known to show substantial correlation [8]) for the 2003 treated data for gradually increasing sample sizes (total number of sequences in this data = 3319). As seen in the figure below, entropy and information estimates improve as sample size increases. Based on this analysis, we chose the NSB entropy estimator for our subsampled datasets of size 300 each.

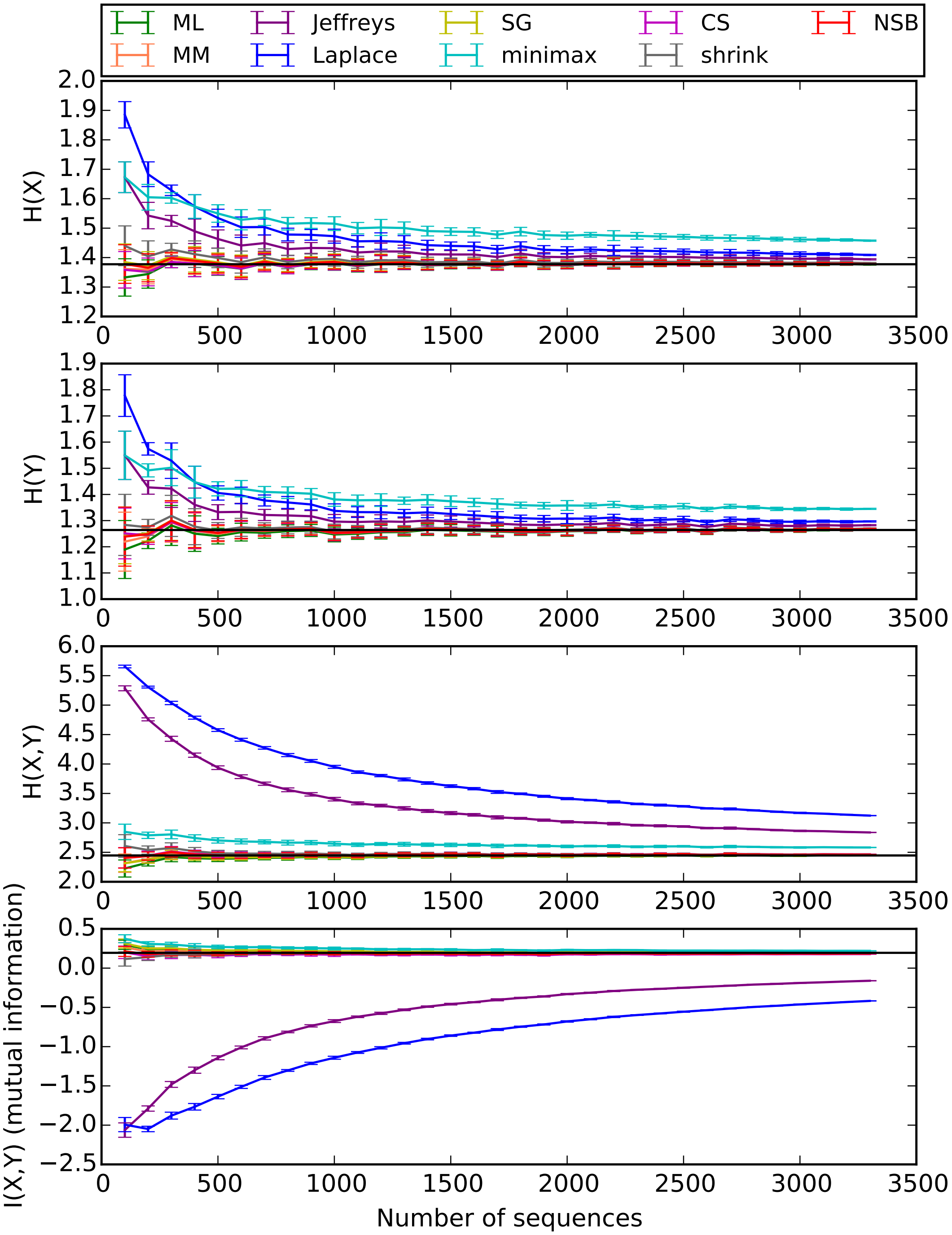

Scaling of entropy and information estimators as a function of sample size. Entropy for positions 54 (X) and 82 (Y) of HIV-1 protease as calculated by the different entropy estimators listed above, for increasing sample sizes (top two panels). The black horizontal lines represent empirical (maximum-likelihood) entropy estimates from all 3,399 sequences, and thus represent the "true" values that the estimators should achieve at smaller sample sizes. The lower two panels show the joint entropy and mutual information estimates for these two positions as a function of sample size. The NSB estimator appears to give the most reliable estimates of entropy and information down to samples as small as 100 sequences.

References

[1] Bialek W. Biophysics: Searching for Principles. Princeton, N.J.: Princeton University Press; 2012.

[2] Miller G. Note on the bias of information estimates. Info Theory Psychol Prob Methods. 1955; II-B:95–100.

[3] Krichevsky RE, Trofimov VK. The performance of universal encoding. IEEE Trans Inf Theory. 1981; 27:199–207.

[4] Schurmann T, Grassberger P. Entropy estimation of symbol sequences. Chaos. 1996; 6:414–427.

[5] Chao A, Shen TJ. Nonparametric estimation of Shannon's index of diversity when there are unseen species in sample. Environ Ecol Stat. 2003; 10:429–443.

[6] Hausser J, Strimmer K. Entropy inference and the James-Stein estimator, with application to nonlinear gene association networks. J Mach Learn Res. 2009; 10:1469–1484.

[7] Nemenman I, Shafee F, Bialek W. Entropy and Inference, revisited. In: Adv Neural Inf Process Syst. vol. 14; 2002. p. 471–478.

[8] Wu TD, Schiffer CA, Gonzales MJ, Taylor J, Kantor R, Chou S, et al. Mutation patterns and structural correlates in human immunodeficiency virus type 1 protease following different protease inhibitor treatments. J Virol. 2003; 77:4836–4847.
