# Supplementary material for: Strong Selection Significantly Increases Epistatic Interactions in the Long-Term Evolution of a Protein
Source: PLoS Genet. 2016 Mar 30;12(3):e1005960. doi: 10.1371/journal.pgen.1005960 (PMC4814079; doi:10.1371/journal.pgen.1005960)
Supplement: S7 Text — (PDF) [file pgen.1005960.s010.pdf]

Supporting Text 7: Three-loci two-allele model for understanding relationship between epistasis and information

To understand the relationship between residue co-variation and epistasis, we constructed a population genetic two-allele three-loci model. With ‘A’ and ‘a’ as the two alleles, the genotypes in the three-loci model are: 1: AAA, 2: AaA, 3: aAA, 4: Aaa, 5: aAa, 6: AAa, 7: aaA, 8: aaa.

If μ is the mutation rate per unit time, then the genotypes are generated from each other according to the figure below.

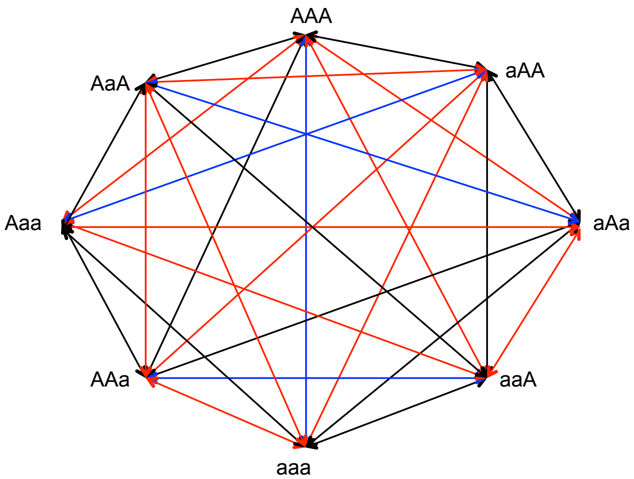

Rates of mutation between the eight different genotypes: black, red, and blue lines indicate that the connected genotypes are accessible by single, double, or triple mutations respectively (*i.e.* μ, μ^2, and μ^3).

The probability to find each of these genotypes in an infinite population depends on the fitness and probabilities of the other genotypes. In a discrete update scheme, the probability to find type i at time $t+1$ is related to the same quantity at time t via

$$p_1^{t+1} = p_1^t \frac{w_1}{\bar{w}} F + \mu \left(\frac{p_2^t w_2 + p_3^t w_3 + p_6^t w_6}{\bar{w}} \right) + \mu^2 \left(\frac{p_4^t w_4 + p_5^t w_5 + p_7^t w_7}{\bar{w}} \right) + \mu^3 \frac{p_8^t w_8}{\bar{w}} \quad (1)$$

$$p_2^{t+1} = p_2^t \frac{w_2}{\bar{w}} F + \mu \left(\frac{p_1^t w_1 + p_4^t w_4 + p_7^t w_7}{\bar{w}} \right) + \mu^2 \left(\frac{p_3^t w_3 + p_6^t w_6 + p_8^t w_8}{\bar{w}} \right) + \mu^3 \frac{p_5^t w_5}{\bar{w}} \quad (2)$$

$$p_3^{t+1} = p_3^t \frac{w_3}{\bar{w}} F + \mu \left(\frac{p_1^t w_1 + p_5^t w_5 + p_7^t w_7}{\bar{w}} \right) + \mu^2 \left(\frac{p_2^t w_2 + p_6^t w_6 + p_8^t w_8}{\bar{w}} \right) + \mu^3 \frac{p_4^t w_4}{\bar{w}} \quad (3)$$

$$p_4^{t+1} = p_4^t \frac{w_4}{\bar{w}} F + \mu \left(\frac{p_2^t w_2 + p_6^t w_6 + p_8^t w_8}{\bar{w}} \right) + \mu^2 \left(\frac{p_1^t w_1 + p_5^t w_5 + p_7^t w_7}{\bar{w}} \right) + \mu^3 \frac{p_3^t w_3}{\bar{w}} \quad (4)$$

$$p_5^{t+1} = p_5^t \frac{w_5}{\bar{w}} F + \mu \left(\frac{p_3^t w_3 + p_6^t w_6 + p_8^t w_8}{\bar{w}} \right) + \mu^2 \left(\frac{p_1^t w_1 + p_4^t w_4 + p_7^t w_7}{\bar{w}} \right) + \mu^3 \frac{p_2^t w_2}{\bar{w}} \quad (5)$$

$$p_6^{t+1} = p_6^t \frac{w_6}{\bar{w}} F + \mu \left(\frac{p_1^t w_1 + p_4^t w_4 + p_5^t w_5}{\bar{w}} \right) + \mu^2 \left(\frac{p_2^t w_2 + p_3^t w_3 + p_8^t w_8}{\bar{w}} \right) + \mu^3 \frac{p_7^t w_7}{\bar{w}} \quad (6)$$

$$p_7^{t+1} = p_7^t \frac{w_7}{\bar{w}} F + \mu \left(\frac{p_2^t w_2 + p_3^t w_3 + p_8^t w_8}{\bar{w}} \right) + \mu^2 \left(\frac{p_1^t w_1 + p_4^t w_4 + p_5^t w_5}{\bar{w}} \right) + \mu^3 \frac{p_6^t w_6}{\bar{w}} \quad (7)$$

$$p_8^{t+1} = p_8^t \frac{w_8}{\bar{w}} F + \mu \left(\frac{p_4^t w_4 + p_5^t w_5 + p_7^t w_7}{\bar{w}} \right) + \mu^2 \left(\frac{p_2^t w_2 + p_3^t w_3 + p_6^t w_6}{\bar{w}} \right) + \mu^3 \frac{p_1^t w_1}{\bar{w}} \quad (8)$$

where $\bar{w}$ is the mean fitness $\bar{w} = \sum_{i=1}^{8} p_i^t w_i$, and F is the fidelity of replication $F = 1 - 3\mu - 3\mu^2 - \mu^3$. It is easy to show that $\sum p_i^{t+1} = 1$ as long as $\sum p_i^t = 1$.

Equations (1-8) can be solved numerically iteratively, but alternatively the fixed point (the p_i in the limit $t \to \infty$) can be calculated by solving for the right eigenvector of the associated Markov matrix.

Armed with the equilibrium probabilities p_i, we can calculate the information between loci as follows. First we define $p(A)$ and $p(a)$ for each of the three loci:

$$\begin{aligned} p^{(1)}(A) &= p_1 + p_2 + p_4 + p_6 \;, \quad p^{(1)}(a) = 1 - p^{(1)}(A) \\ p^{(2)}(A) &= p_1 + p_3 + p_5 + p_6 \;, \quad p^{(2)}(a) = 1 - p^{(2)}(A) \\ p^{(3)}(A) &= p_1 + p_2 + p_3 + p_7 \;, \quad p^{(3)}(a) = 1 - p^{(3)}(A) \end{aligned} \quad (9)$$

giving us the marginal entropies of the first and second locus

$$\begin{aligned} H(1) &= -\sum_{i=a,A} p^{(1)}(i) \log p^{(1)}(i) \;, \\ H(2) &= -\sum_{i=a,A} p^{(2)}(i) \log p^{(2)}(i) \;, \\ H(3) &= -\sum_{i=a,A} p^{(3)}(i) \log p^{(3)}(i) \;. \end{aligned} \quad (10)$$

The joint entropies for the pairwise loci are:

$$\begin{aligned} H(1,2) &= -\sum_{i=a,A} \sum_{j=a,A} p^{(1,2)}(i,j) \log p^{(1,2)}(i,j) \;, \\ H(1,3) &= -\sum_{i=a,A} \sum_{j=a,A} p^{(1,3)}(i,j) \log p^{(1,3)}(i,j) \;, \\ H(2,3) &= -\sum_{i=a,A} \sum_{j=a,A} p^{(2,3)}(i,j) \log p^{(2,3)}(i,j) \;. \end{aligned} \quad (11)$$

The shared entropies (or information) are

$$I(1:2) = H(1) + H(2) - H(1,2) \;,$$

$$
\begin{aligned}
I(1:3) &= H(1) + H(3) - H(1,3)\ , \\
I(2:3) &= H(2) + H(3) - H(2,3)\ .
\end{aligned} \tag{12}
$$

Naturally, the sum over pairwise mutual entropies $MI = I(1:2) + I(1:3) + I(2:3)$. Three-way epistasis in a two-allele three-loci model is defined as:

$$
E = \log\left(\frac{w_{AAA} \times w_{aaa}}{w_{AaA} \times w_{aAA} \times w_{Aaa} \times w_{aAa} \times w_{AAa} \times w_{aaA}}\right)\ . \tag{13}
$$

The simulation is run for 4000 updates, with the fitness landscape changing at the 2000th update as follows: starting with only the wild type genome as the most fit ($w_{AAA} = 1.0$), the fitness landscape becomes more rugged as the triple mutant become as fit as wild-type ($w_{AAA} = w_{aaa} = 1.0$). The fitnesses of the intermediate genotypes remain close to zero (10^{-5}) throughout the simulation.

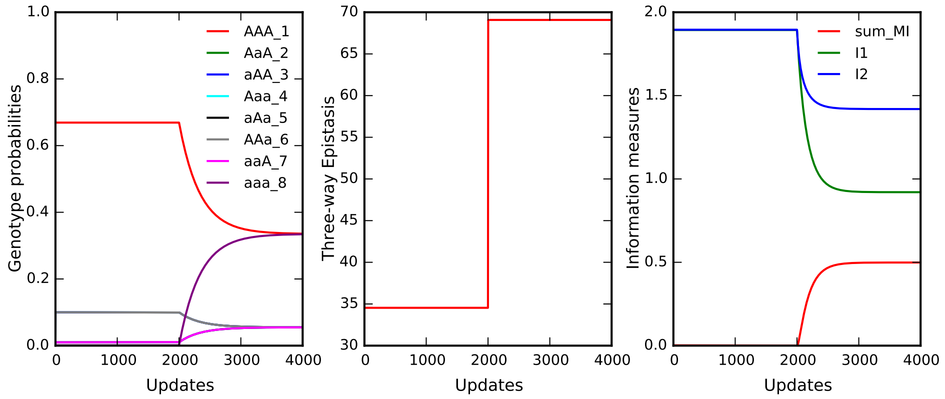

Three-loci two-allele simulation. Left panel shows the changes in genotype probabilities as the triple mutant (aaa) becomes as fit as the wild-type (AAA) at the 2000th update. Middle panel shows the increase in the three-way epistasis as the landscape becomes more rugged. Right panel shows the information measures: I_1, I_2, and sum of pairwise mutual information (MI). Notice that sum of MI increases with increase in epistasis.
